# Long-Term Effects of SARS-CoV-2 in the Brain: Clinical Consequences and Molecular Mechanisms

**DOI:** 10.3390/jcm12093190

**Published:** 2023-04-28

**Authors:** Ann-Charlotte Granholm

**Affiliations:** Department of Neurosurgery, University of Colorado Anschutz Medical Campus, Denver, CO 80045-0511, USA; lotta.granholm@cuanschutz.edu

**Keywords:** SARS-CoV-2, COVID-19, neurodegeneration, Down syndrome, concussion, neurotrauma, infectious disease and brain

## Abstract

Numerous investigations have demonstrated significant and long-lasting neurological manifestations of COVID-19. It has been suggested that as many as four out of five patients who sustained COVID-19 will show one or several neurological symptoms that can last months after the infection has run its course. Neurological symptoms are most common in people who are less than 60 years of age, while encephalopathy is more common in those over 60. Biological mechanisms for these neurological symptoms need to be investigated and may include both direct and indirect effects of the virus on the brain and spinal cord. Individuals with Alzheimer’s disease (AD) and related dementia, as well as persons with Down syndrome (DS), are especially vulnerable to COVID-19, but the biological reasons for this are not clear. Investigating the neurological consequences of COVID-19 is an urgent emerging medical need, since close to 700 million people worldwide have now had COVID-19 at least once. It is likely that there will be a new burden on healthcare and the economy dealing with the long-term neurological consequences of severe SARS-CoV-2 infections and long COVID, even in younger generations. Interestingly, neurological symptoms after an acute infection are strikingly similar to the symptoms observed after a mild traumatic brain injury (mTBI) or concussion, including dizziness, balance issues, anosmia, and headaches. The possible convergence of biological pathways involved in both will be discussed. The current review is focused on the most commonly described neurological symptoms, as well as the possible molecular mechanisms involved.

## 1. Introduction

Viruses of the *Coronaviridae* family are single-stranded RNA viruses [1]. They are broadly found in different animal species, including cats, dogs, birds, bats, and mice. Some coronaviruses are pathogenic to humans and may cause severe respiratory disease, such as severe acute respiratory syndrome (SARS) and Middle East respiratory syndrome (MERS), while others do not infect humans. Severe acute respiratory syndrome coronavirus 2 (SARS-CoV-2) is a coronavirus that has created chaos in the world since late in the year 2019 [2] and gives rise to the disease commonly called COVID-19 (named by the WHO as the coronavirus disease of 2019). For simplicity, we will use the term COVID-19 for the disease caused by the virus and SARS-CoV-2 for the virus itself in this review. To date, close to 700 million cases have been reported worldwide, and the numbers continue to rise. Worldwide deaths related to COVID-19 have been reported to be more than 6.8 million to date. The Worldwide Health Organization (WHO) declared the outbreak a public health emergency of international concern in January of 2020 and a pandemic in March of 2020. In the latter part of 2020, the epidemiology, pathophysiology, and clinical symptomology of patients affected by a SARS-CoV-2 infection revealed that patients with Down syndrome (DS), congenital heart disease, chronic kidney disease, obesity, airway obstruction, COPD, and pulmonary hypertension were at higher risker for serious complications [3,4,5,6] (see also www.cdc.gov accessed on 3 March 2023).

Recent investigations have demonstrated several viral infections that can be involved in the onset or progression of neurological diseases (see, e.g., [7,8]). Neurodegenerative conditions, including amyotrophic lateral sclerosis (ALS), Parkinson’s disease (PD), Alzheimer’s disease (AD), and multiple sclerosis (MS), often have multifactorial etiology, including environmental and genetic components acting in concert. However, both viral and bacterial toxins that affect the central nervous system (CNS) can contribute significantly to neurodegeneration, as evidenced by the experiments first performed in rats [9]. In that study, the investigators injected one single dose of tetanus toxin, a bacterial toxin, into the hippocampus of rats and found a time-dependent degeneration of the hippocampal neurons, suggesting a direct effect of bacterial toxins on these cells that resulted in progressive neurodegeneration.

**Figure 1:**
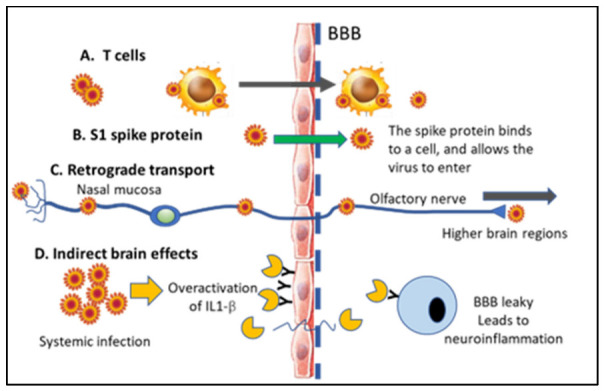
**Direct and indirect effects of SARS-CoV-2 virus on the brain and mode of entry into the CNS.** (**A**) The virus can enter together with T cells since an infection can alter the BBB permeability. (**B**) The SARS-CoV-2 Spike 1 protein can attach to endothelial cells and insert the virus into the cells of the BBB. (**C**) The virus attacks the nasal mucosa and can be transported retrogradely via olfactory nerves either with direct retrograde transport or via exosomes into the CNS. The same retrograde transport of viral particles has also been proposed in other cranial nerves (trigeminal, facial, and vagal nerves). (**D**) Indirect entry of the SARS-CoV-2 virus into brain tissue may be caused by overactivation of the IL1-β inflammatory system in the body after infection, leading to elevated IL-1β and other proinflammatory cytokines in the blood. IL-1β has receptors on endothelial cells, and binding to its receptors causes re-conformation of the BBB, allowing both blood cells and cytokines to enter the brain. IL1-β then binds to its receptors on glial cells and neurons within the CNS, leading to progressive neuroinflammation and neuronal loss. Since brain pathology is caused by blood-based cytokines and not the virus itself, this leads to an indirect effect on the brain via systemic infection. Small solar-shaped figure represents the virus and yellow shape in D represents IL-1β. “Y” in D represents IL-1β receptor.

There can be either direct neurological effects caused by the SARS-CoV-2 virus entering the brain or spinal cord or indirect effects caused by the immune response in the body and/or in the brain that can trigger pathology in the CNS (Figure 1) (see also [10,11]). There are two proteins that are particularly involved in the infection of cells by the SARS-CoV-2 virus. First, SARS-CoV-2 enters recipient cells by the binding of its spike protein to the angiotensin-converting enzyme 2 (ACE2) receptor, which is expressed on the cell membrane of many cell types [12]. ACE2 has been shown to be expressed by neurons, astrocytes, oligodendrocytes, and epithelial cells in the brain [13,14,15,16] and also in microglial cells [17,18]. Interestingly, in a recent study by Lindskog and collaborators [19], an upregulation of ACE2 was observed in endothelial cells in the brains of patients with COVID-19, most prominently in the white matter and with the highest expression observed in the patients exhibiting the most severe neurological symptoms [19].

Clough and collaborators [17] suggested that the virus activates a mitochondrial-dependent intrinsic apoptotic pathway, which then results in apoptosis [17]. These investigators showed that administration of the SARS-CoV-2 spike protein gave rise to increased apoptosis of cultured human microglial cells. This could explain the finding that there are fewer microglial cells in the gray matter of *postmortem* brain tissue obtained from patients infected with SARS-CoV-2, which might then suggest that COVID-19 neurological symptoms are mediated at least partially by microglial cell loss [20], leading to less ability to handle the cytokine storm ensuing after infection. It has also been shown that the SARS-CoV-2 virus can spread to the brain via the olfactory bulb and that infection in the brain spreads via neuron-to-neuron transmission, leading to neuronal cell loss in the areas afflicted [21]. These investigators also showed that intracranial inoculation, even with low doses of the virus, resulted in a lethal disease, even though little infection was detected in the lungs of mice injected with SARS-CoV-2 virus [21]. Others have demonstrated reduced hippocampal neurogenesis in mice treated with the SARS-CoV-2 virus, even after a mild respiratory infection [22]. Thus, several cell types in the brain, including neurons, are involved in the rapid onset of neurological symptoms in some people with COVID-19.

Transmembrane serine protease 2 (TMPRSS2) is a cell surface serine protease that is also required for the virus to enter the cell. Both ACE2 and TMPRSS2 are expressed in the nasal and respiratory epithelium [23,24]. TMPRSS2 is known to prime the spike protein domain, which leads to the virus fusing to epithelial cells by binding to ACE2, and for priming the spike domain of SARS-CoV-2 by cleaving at the S1/S2 sites [16,23]. When SARS-CoV-2 binds to the ACE2 receptor, the bioavailability of the virus becomes limited, which induces an injury/inflammatory cascade in the cell. This leads to a cytokine storm that affects both tissues in the body and the brain. In tissue culture, cells that express higher levels of TMPRSS2 also yield higher SARS-CoV-2 particle counts, leading to increased toxicity [25,26]. TMPRSS2 serves as a cofactor for SARS-CoV-2 cell entry and can prime glycoproteins of other respiratory viruses as well [27]. Due to its role in cell entry, TMPRSS2 has been a target for drug development to combat coronaviruses with some success in the last few years [25]. TMPRSS2 can increase the entry efficiency, and therefore, this protease plays a major role in the infection rate and severity and may be easier to target for drug interventions than the ACE/ACE2 system.

Activation of the inflammatory processes, as well as the host immune response, can cause chronic damage resulting in misfolded proteins, neuronal loss, chronic microglial activation, and progressive degenerative diseases, including those mentioned above (see, e.g., [8]). These studies clearly suggest that viral or bacterial infections can have serious consequences not only in the body but can also affect pathological processes in the brain and spinal cord and contribute to the initiation or progression of neurodegenerative conditions. Neurovirology has emerged over the last couple of decades and is a relatively new discipline of neuroscience but an important one in view of the recent pandemic. The current review is focused on neurological symptoms and biological mechanisms for SARS-CoV-2 effects on the brain.

As the pandemic continued to rage in the late summer and fall of 2020, a new set of symptoms was discovered and reported in the literature—neurological side effects of the primary viral infection caused by SARS-CoV-2. Investigators found that even younger persons could present with severe neurological symptoms, including paresthesia, depression, dysautonomia [28], and brain fatigue, while older persons were more likely to experience encephalopathy, seizures, encephalitis [29], delirium, or worsening of an ongoing dementia [30] (see also Table 1 below).

Certain patient groups among older adults appeared to be especially sensitive toward COVID-related comorbidities and mortality. In fact, it has been shown that patients with a comorbidity of Alzheimer’s disease (AD) and COVID-19 exhibit the highest mortality risk among older adults, while those with a preexisting diagnosis of Parkinson’s disease (PD), another common neurodegenerative disease, have a higher risk than the general population of contracting COVID-19 but not a higher mortality in an investigation that included more than 13,000 older adults in the UK [31]. These findings are interesting and suggest that there are features of AD, or other CNS pathologies, that lead to increased vulnerability toward SARS-CoV-2. These are discussed in more detail below. Viral infections thus appear to be involved in the progression of AD pathology in the brain, opening up a whole new field of research into neurovirological mechanisms for AD [32,33].

It is also shown that SARS-CoV-2 infections can result in autoimmune disorders [29,34,35].

Viral infections may trigger an autoimmune disease, especially in COVID-19 patients who are already at risk of developing autoimmune conditions [34]. Certain autoimmune diseases affect the brain and spinal cord [36] and therefore indirectly result in neurological symptoms post-COVID. Autoimmunity can happen during a hyper-stimulated state of the immune system and could be due to molecular mimicry between pathogen and host proteins. It has been shown previously that viruses can contribute to the production of autoimmune antibodies and lead to the development of autoimmune diseases [37]. Autoimmune manifestations of SARS-CoV-2 have been reported and can contribute to the neurological symptoms associated with this virus. Al-Beltagi and collaborators [35] suggested that one potential mechanism for the development of autoimmune diseases could be the binding of the SARS-CoV-2 spike protein to the ACE2 receptor. This may cause long-term disabilities for a group of vulnerable patients reacting to the viral infection with a sustained inflammatory reaction. In addition, immunomodulatory or immune-suppressive drugs such as IL-1β receptor blockers that are already FDA approved could represent likely candidates for COVID-19 therapy (see, e.g., [38]).

Interestingly, coronaviruses in general were considered to give rise to modest respiratory illnesses until the discovery of severe acute respiratory syndrome (SARS) caused by SARS-CoV in 2002, followed by the Middle East respiratory syndrome (MERS) caused by MERS-CoV in 2012 [39]. SARS and MERS induced severe viral pneumonia with respiratory failure in some cases but were also ascribed to numerous extrapulmonary manifestations, including neurological symptoms [40]. There is reason to believe that these coronaviruses are neuroinvasive and can therefore cause severe detrimental effects on neurons in the brain and spinal cord [41,42,43,44].

Desforges and collaborators [42] speculated that human coronaviruses are neurovirulent, since SARS and MERS, as well as other viruses affecting humans, are known to contribute to short- and long-term neurological manifestations as well [45,46]. Viruses can enter the CNS either via hematogenous transport or via retrograde axonal transport [44]. Since SARS, MERS, and SARS-CoV-2 are respiratory viruses, which enter the body via aerosols, it is likely that they may enter the CNS via nerve terminals located in the upper respiratory tract, the olfactory mucosa, which represents the port of entry to the brain via the olfactory bulb and the first cranial nerve. Viral particles of SARS-CoV-2 have been discovered in *postmortem* tissues of the brain, both in the cerebrospinal fluid (CSF) and in the gray matter [43,47,48,49,50,51]. This could explain the symptoms of SARS-CoV-2 infections, such as loss of smell and taste, headaches, fatigue, nausea, and dizziness.

It is important to keep in mind that *postmortem* examination of the brain tissue was mostly conducted in persons with severe COVID-19 infection who died from the disease, so it may not necessarily be true for all persons who were infected with this virus. Other coronaviruses have also been detected within the CNS in humans, including SARS [52]. The initial symptoms of SARS included nausea, dizziness, fever, confusion, and, in about 20% of the cases, severe respiratory illness [53]. People who contracted MERS had similar extrapulmonary symptoms, including several neurological manifestations [40].

The mortality rates for SARS and MERS were 10% and 35%, respectively, while the mortality rate for COVID-19 is an average of 5.6% [54]. All three infections (SARS, MERS, and COVID-19) give rise to a broad spectrum of illnesses, including asymptomatic, mild, or severe multisystem symptoms. It should be noted, though, that mortality, as well as the array of common symptoms, will change depending on which strain of the virus is being referred to and the proportion of individuals in a state or region that are currently vaccinated. This is a complex pandemic with multiple factors affecting the outcome.

The well-described findings regarding neurological disease and symptoms following SARS and MERS can be used to draw potential scenarios as to what will happen with COVID-19 patients in the long term. Unfortunately, COVID-19 has killed more people than the SARS and MERS epidemics combined due to the significant spread of the disease worldwide [55]. Therefore, it is of utter importance to investigate the long-term neurological effects of the SARS-CoV-2 virus in brain tissue and the spinal cord since this will be an emerging healthcare issue. This is the focus of the current review, albeit with several effective vaccines distributed currently.

## 2. Common COVID-19-Related Neurological Symptoms

Soon after the beginning of the pandemic, it became evident that there would be more than just acute medical pulmonary or cardiovascular problems associated with SARS-CoV-2 infections. As discussed above, the neurological and neuropsychiatric manifestations have also been widely seen with other viral infections [40,45]. Neurological symptoms and encephalopathy were reported in numerous patients, not only those with severe but also with milder symptoms of SARS-CoV-2 infections [56,57]. The most common neurological complications include anosmia and hypogeusia (loss of smell and taste), dizziness, headaches, general fatigue, seizures, and stroke, as well as delirium and balance disturbances (see Table 1 and [58]).

A positron emission tomography (PET) imaging study was conducted on patients with at least 3 weeks post-infection of functional complaints [56]. Hypometabolism was observed in long COVID cases in the orbital gyrus, which includes the olfactory gyrus and the right temporal lobe, including the amygdala and the hippocampus, brainstem, and cerebellum [56]. These fluorodeoxyglucose (FDG) PET findings were highly discriminatory between long COVID patients and controls.

In terms of stroke in patients with COVID-19, specific disease features have been reported, with stroke being commonly reported in younger patients without the more classical risk factors observed for stroke [59]. In a study from Thomas Jefferson University, stroke patients who had COVID-19 were often younger, had multiple large vessels blocked, and had a worse outcome than stroke patients without a history of SARS-CoV-2 infection [59], suggesting that the virus affects clotting even without the common risk factors observed. A high prevalence of cryptogenic stroke, as well as increased risk for large vessel stroke, have been noted even in patients with reportedly milder cases of the infection (see Table 1). These findings strongly suggest that one must be aware of the stroke risk also caused by the SARS-CoV-2 infection in younger patients [60] and that vascular blockage or bleeds are common features of the consequences of this virus.

**Table 1:** **Common neurological symptoms/conditions associated with SARS-CoV-2**.

Symptom	Age Range	Citation
Anosmia or hypogeusia	All ages	[41,57,58,61,62]
Encephalopathy	Older adults	[29,63]
Balance disturbances and limb force reductions	All ages	[58]
Cognitive impairment	60 and older	[56,64]
Seizures	Older adults	[44,48]
Hemorrhagic events, including CVST	Females < 50 years old	[40,48,58]
Stroke (most common: cryptogenic stroke)	<55 years old	[59,60]
Delirium	Older adults/dementia	[65]
Severe headaches	14–60% of all patients	[66]
General fatigue	All ages	[67]

Severe migraine-like headaches are common in both the early phases of COVID-19 and in long COVID [41,45,48,66]. The headaches associated with acute infection are reported as oppressive pain in the frontal portion of the head and affects between 14 and 60% of adult patients during the acute COVID-19 phase [45,48]. This is often the earliest symptom of COVID-19. One potential reason for the early involvement of the frontal lobe is the route of infection described for coronaviruses via the nasal epithelium and the olfactory bulb [13] (see above). Headaches are also common in children afflicted by a SARS-CoV-2 infection [68]. Severe headaches have been reported to be a crucial part of long COVID, a condition that affects the ability to work and quality of life-long term in adults of all ages. The symptoms and clinical comorbidities of long COVID are sometimes difficult to recognize [69].

Patients have reported intermittent but deep, stabbing, tension-like or migraine-like headaches for months following an acute infection. It is important to be cautious about secondary headaches in long COVID, as these could be caused by cerebrovascular complications, including stroke or microbleeds, and need to be differentiated from other primary and secondary headache disorders. It has been shown that long COVID can be associated with headache episodes that are clinically indistinguishable from migraines even in patients without a history of migraines [66,69]. Daily headaches can give rise to functional impairment and psychological or psychiatric comorbidities. Headaches associated with either acute or long COVID are caused by hypoxia, vascular events, or ongoing immunologic events, as discussed by others [70]. Due to increased survival from COVID-19 from widespread vaccination practices, long COVID has emerged as an increasing medical and socioeconomic problem in the Western world.

Interestingly, some neurological symptoms have also been reported, albeit rarer, after vaccination against the SARS-CoV-2 virus. These include ischemic and hemorrhagic stroke, cerebral venous sinus thrombosis (CVST), encephalopathy, Ball’s palsy, and Guillain–Barre syndrome (GBS) [71]. Most of the vaccines that have been developed for SARS-CoV-2 target the viral spike proteins by generating antibodies to prevent infection. Some of the vaccines are known to cause more side effects than others and have therefore not been commonly approved. The vaccine platforms used for SARS-CoV-2 include whole virus vaccines, viral vector vaccines, and nucleic acid vaccines (RNA and DNA), as well as hybrid forms thereof [72]. The risk for developing neurological complications following vaccination is small compared to the complications observed when unvaccinated patients are infected [73]. Vaccines are not the main focus of the current review and have been reviewed in detail elsewhere [74] but nonetheless represent an area of intense debate, unfortunately allowing vaccination practices to be the target of political campaigning.

## 3. Cognitive Impairment and Alzheimer’s Disease

Cognitive impairment, dementia, and “brain fog” have been reported in severe COVID-19 patients [64,75]. Dementia is classified as progressive or persistent loss of intellectual ability, including impairment of memory and abstract thinking, executive function, decision making, aphasia, and/or personality change [30]. Potential pathophysiological mechanisms for cognitive impairment associated with either acute or long COVID include neuro-invasion via cranial nerves, such as the olfactory, trigeminal, optic, and vagal nerves. The cytokine storm that might affect the blood–brain barrier (BBB) may lead to the invasion of proinflammatory cytokines from the blood into brain structures that are sensitive to infection, such as the medial temporal lobe, as discussed in more detail in Figure 1. There are common risk factors associated with severe COVID-19 and Alzheimer’s disease (AD).

For example, ACE2 converts angiotensin I to angiotensin 1–9 and serves as a receptor for entry of the SARS-CoV-2 virus, as described above [23,24,76,77,78], and the ACE2 levels are elevated in the hippocampus of patients with AD [14]. Ang-II exhibits harmful effects on the brain, including vasoconstriction and proinflammation, while Ang (1–7) has the opposite effects and exerts mild vasodilation, along with protection against cardiovascular disease [79]. The relationship between ACE2 and AD is complicated, since other studies show that reduced levels of ACE2 lead to cognitive impairment in mouse models [80]. For example, recent studies have shown that Aβ42 is converted to the less harmful Aβ40 by ACE and ACE2 [81], demonstrating direct effects of this receptor on the aggregation properties of amyloid in AD. Since there are drugs available that regulate ACE2 activity and/or levels [82], this is a road forward towards effective interventions against SARS-CoV-2 infections. Thus, ACE2 activity and levels increase SARS-CoV-2 infection, while ACE2 expression decreases once viral infection occurs. The controversy in this field could be due to differences in experimental methods and models, as well as dual functions of ACE2 receptors and the angiontensins, and the field will benefit from more mechanistic studies.

Another potential risk factor for AD in the COVID-19 pandemic is the ApoE genotype. The *APOE* gene codes for a protein called apolipoprotein E, which combines with lipids to form lipoproteins. ApoE is involved in the packaging and transportation of cholesterol, and there are at least three different isoforms of the *APOE* gene product, including ApoE2, -3, and -4 [83]. The ApoE4 genotype is the strongest susceptibility factor for sporadic AD [83], but the ApoE4 genotype is also associated with the occurrence and severity of infections, for example, with the following viruses: HIV-1, herpes simplex, and hepatitis C [84]. Cohort studies indicate that having the ApoE4 genotype can exhibit as much as a two-fold increased risk in mortality for COVID-19 [84], providing evidence that susceptibility toward severe infection outcomes also exist for this virus. The mechanisms for ApoE4 involvement in long COVID are interesting [85] and deserve heightened focus considering the pandemic and the severe outcome for AD patients.

These findings show that AD results in increased mortality for COVID-19, and there are now known risk factors that can affect the outcome. The next steps in this field could be to identify effective treatment paradigms that may target ACE2 and/or other mechanisms involved in increased severity of infection for the AD patient population.

## 4. COVID-19 and Delirium

An unfortunate extrapulmonary consequence of severe COVID-19 in older adults, especially those with comorbidities such as AD, is delirium. Delirium results in confused thinking and a lack of awareness of day and time and is often confused with AD. Delirium usually comes on fast, within hours or a few days. In a recent study by Tyson et al. [65], they examined delirium in patients who came into the emergency department (ED) with severe COVID-19. The delirious group could be distinguished from the non-delirious group, as they were more likely to have neurologic, psychiatric, or cardiovascular comorbidities, as well as using deliriogenic medications and/or with a prior history of delirium. Previous studies have shown that delirium predicts mortality and occurs more often in older than in younger adults. Tyson et al. further reported that delirium related to COVID-19 had a highly significant correlation with neurological comorbidities and dementia. Stroke also correlated with delirium to a significant degree (*p* < 0.002) [65]. Of the more than 500 patients examined in the Tyson study, only about 13% met the diagnostic inclusion criteria for delirium. Thus, this study suggested that delirium is not one of the most common neurological consequences of COVID-19, but one should nonetheless pay attention to this serious condition developing, as delirium correlates with serious outcomes, including mortality [86]. Relatively little attention has been paid to the prevalence or consequences of delirium in patients with severe COVID-19, even though other studies indicated a prevalence of delirium in as much as every third patient with severe COVID-19 [86].

## 5. Increased Vulnerability in Patients with Traumatic Brain Injuries

An exaggerated response to SARS-CoV-2 has been reported in individuals who have had one or repeated mild traumatic brain injuries (mTBIs) or concussions (Prusmack, oral communication). As mentioned above, neurological symptoms seen in individuals with post-concussive syndrome (PCS) are strikingly similar to neurological symptoms occurring in COVID-19, either in acute or long COVID cases, and include headaches, dizziness, and problems with concentration and memory [87]. A recent study by Bullock and collaborators [88] showed that high school athletes who had an active COVID-19 infection were three times more likely to sustain a concussion. This may be due to deconditioning because of reduced training habits during illness but could also potentially be due to parallel pathophysiological events in the brain. Similar to what is seen in the brain post-COVID, an mTBI also results in an insult on endothelial cells involved in the BBB [89]. While the breakdown of the BBB observed in COVID-19 seems to primarily be caused by cytokine-related conformational changes [90], mTBIs also give rise to physical damage to endothelial cells, i.e., a primary injury, followed by cytokine-related alterations, a secondary injury, caused by an inflammatory cascade post-injury. Interestingly, the biomarkers for TBI are elevated in the plasma of severe COVID-19 cases [91]. Since inflammation is a key player in neurotrauma [92], it is possible that persons who have already sustained one or repeated mTBIs are especially vulnerable to COVID-19 infections due to a lingering widespread neuroinflammation in the brain. These findings may result in anti-inflammatory treatment as a prevention for COVID-related neurological symptoms.

## 6. Molecular Mechanisms for Brain and Cell Entry

### 6.1. Brain Aging and Aggregation of Misfolded Proteins

Some studies have suggested that the SARS-CoV-2 virus accelerates aging in the brain [93]. Accelerated aging can be caused either by genetics, such as with persons with progeria or with Down syndrome (DS), or environmental factors, including repeated or severe infections [94]. Senescent cells stop dividing and shift their phenotype towards survival rather than development, which leads to a senescence-associated secretory phenotype (SASP) [94]. Senescence leads to altered morphology and proteostasis, impairment of the lysosomal pathways, including autophagy, and the accumulation of lipid droplets known as lipofuscin in the brain. With aging in the brain, epigenetic alterations, including DNA methylation, chromatin remodeling, and histone post-translational modifications, are known to occur and collectively lead to reduced function and progressive neuronal loss. According to Mattson et al. [95], the hallmarks of brain aging include reductions in neuronal plasticity, as well as dysfunctional neuronal network activity, increased oxidative stress, and inflammation. Interestingly, many of these “brain aging” hallmarks are also observed in patients of all ages with severe COVID-19. For example, Karnik and collaborators reported neuroinflammation as a hallmark for brain involvement of COVID-19 [96], and investigators have reported multiple biomarkers associated with AD and neurotrauma in patients with severe COVID-19 [75]. Other hallmarks of aging reported for COVID-19 include an overactive systemic immune response, ischemia/hypoxia or disruption of perfusion in the brain, and hyperactivation of astrocytes in brain parenchyma [97]. In a recent study published in *Nature* by Mavrikaki [98], the investigators performed an RNA study on patients with different severities of COVID-19 infections. They found thousands of differentially expressed genes (DEGs) in the patient samples compared to the controls. Upregulated DEGs were enriched for genes involved in immune-related pathways, and downregulated DEGs were enriched for genes involved in learning and memory, as well as synaptic plasticity and activity [98]. This is a genetic profile that resembles those previously observed in aging brains; hence, the investigators stated that COVID-19 may result in accelerated brain aging.

Others have also suggested that mental effects in severe COVID-19 patients mimic the cognitive capacity reduction observed with aging. This is consistent with other findings, showing that the SARS-CoV-2 virus can induce the aggregation of misfolded proteins [99,100], including alpha-synuclein, phospho-Tau and amyloid, also a common feature of the aging brain [101]. The misfolding of proteins, leading to neurodegeneration, can be the result of impaired adaptive stress response and/or oxidative stress, impaired DNA repair and autophagy, and neuroinflammation [95]. Thus, molecular, imaging, and psychiatric investigations all point to an accelerated aging profile in the brain with long COVID, including reduced cognitive capacity, dysregulated immune response, and the aggregation of toxic proteins/neurodegeneration [56,75,93]. Recognizing these pathways might allow for drug interventions, as well as lifestyle factors, that are effective in also promoting healthy aging for long COVID patients.

### 6.2. Direct vs. Indirect Effects in the CNS and Presence of the SARS-CoV-2 vs. in Neurons

Studies have suggested at least three different entryways in which the SARS-CoV-2 virus can enter the CNS [24], as discussed in Figure 1. The SARS-CoV-2 coronavirus invades the CNS via neuronal and hematogenous routes, in addition to viral infection in peripheral tissues with extensive crosstalk with the CNS [60]. This includes retrograde transport via, for example, the sensory axons in the nasal mucosa [102], vascular entryways, and direct entry via the blood–brain barrier (BBB) [24]. Although it is clear that the SARS-CoV-2 virus enters brain parenchyma and causes neurological symptoms, there is less discussion in the literature regarding whether only certain brain regions are afflicted or how the virus may spread from region to region in the brain. There have only been a few histopathological studies conducted on brain donations from COVID-19 victims.

Heppner and collaborators published the first evidence showing SARS-CoV-2 virus entry into neurons in the CNS [47]. They discovered SARS-CoV-2 immunostaining in sensory neurons of the olfactory mucosa and suggested that this was one of the entryways for the virus into the CNS and that this could also explain the anosmia and hypogeusia, which are common symptoms of the condition [41,102,103,104]. In a recent study [57], we confirmed that loss of smell and/or taste were indeed only observed in individuals who tested positive for COVID-19 in an antibody test [57]. The intranasal administration of SARS-CoV-2 in mice leads to rapid accumulation in the brain, with the frontal cortex being affected first [47,105]. Song et al. [105] showed, using several different methods, entry of the virus into the neurons, for example, using organoids, as well as a mouse model overexpressing ACE2. In addition, they demonstrated that the infection of neurons with the virus could be blocked by ACE2 inhibitors or the administration of CSF from patients with COVID-19 [105].

As discussed above, ACE2 and TMPRSS2 are abundantly present in the olfactory epithelium and in neurons [16,23,82,106]. The expression of these two entry proteins is higher in aged mice and humans, which could explain the increased sensitivity to the virus with increasing age [23,107]. In addition, there are other mechanisms, yet unknown, by which the virus could enter the CNS and cause neurological symptoms [108]. ACE2 downregulation and a disproportionate response of the immune system may also contribute to multiple organ injury in COVID-19, including the brain [78,109].

Which brain regions are most affected by SARS-CoV-2 infection is still debated. However, the encephalitis seen in many patients with severe COVID-19 suggests focal brain injuries, which might be reversible and connected to acute or chronic vascular changes in the brain. Although relatively few pathology studies have been published to date, the findings in human brains show that the viral infection in the brain is associated with alterations in both neurons and glial cells, particularly the glia lining of cerebral blood vessels [76]. It has been shown, using mouse studies, that the SARS-CoV-2 virus can persist in brain parenchyma for many months following an acute infection. In humans, significant brain swelling has been reported, even in young patients [110]. In particular, the hypothalamus and the cerebellum contained virus particles, along with the frontal cortex and areas associated with sensory input, such as the thalamus and the olfactory bulb. In a study by Agrawal et al. [110], they examined 20 donated brains from individuals with severe COVID-19 who died at the ages of 26–97. They described both acute and chronic alterations in brain health. More than 50% of the brains showed microscopic or macroscopic vascular lesions, regardless of preexisting conditions. These included hemorrhagic and ischemic vascular lesions, as well as microglial activation in the white matter, and T-cell invasion, as well as regional neuronophagia in the locus coeruleus (LC), the medullar inferior olivary nucleus, and cerebellar dentate nucleus in some of the cases, and microglial nodules were observed in particular in the brainstem [110]. It is interesting that the LC is affected by COVID-19, since some of the earliest symptoms in long COVID were the lack of attention and reduced working memory—brain functions that are affected by LC activity [111]. In the patients over 65, chronic vascular and/or neurodegenerative changes were observed, fitting with the hypothesis described above regarding the SARS-CoV-2 virus causing accelerated brain aging. Although they were unable to find viral particles in the brain, a collaborator at the University of Lund (Dr. Elisabet Englund) has described the presence of viral dsRNA in the vascular wall, as well as perivascular inflammation in *postmortem* materials from infected patients [112]. In addition, Song and collaborators [105] also detected the SARS-CoV-2 spike protein in different brain regions in *postmortem* brain tissue in patients with the severe disease. They described the presence of SARS-CoV-2 spike protein immunostaining in several brain regions, including the caudate and BA6, severe global encephalopathy, and diffuse microbleeds, with spike protein staining seen in the periphery of the bleeds [105]. In another *postmortem* study [113], the investigators reported neuro-invasion in the following brain regions after SARS-CoV-2 infection: cranial nerve nuclei, olfactory bulb, motor nuclei of the vagus nerve, and the trigeminal nerve nuclei, as well as the choroid plexus, leptomeninges, median eminence of the hypothalamus, and area postrema of the medulla [113]. The subject with encephalitis in that study was SARS-CoV-2 immunopositive in the entorhinal cortex. The areas of the brain affected by neuro-invasion are thus widely different between different studies, and a more thorough investigation of the neurotropism of this novel virus is therefore needed. In addition, there are widely different opinions regarding the ability of the virus to enter neurons and remain in neurons post-acutely after a systemic infection—something that will no doubt be a focus for continued and expanded investigations. It is likely that these widely different results from different labs could be due to different experimental models, fixations, or the examination of patients at different times after acute infection.

Recent studies showed that patients with metabolic syndrome (overweight, diabetes, and/or hypertension) experience a particularly bad outcome if infected with SARS-CoV-2 [114]. This could be explained by an overactivation of the Interleukin-1 beta (IL-1β) system in these conditions (see Figure 1). This proinflammatory cytokine is elevated in the plasma of those with metabolic syndrome [115], and IL-1β receptors are located on BBB endothelial cells as mentioned above [89]. The binding of IL-1β to its receptors on endothelial cells in the brain results in alterations in the permeability of the BBB, allowing proinflammatory cytokines and immune cells entry to the brain parenchyma [116]. Since neurons and the glia have IL-1β receptors [117], this can lead to an accelerated neuroinflammation, which is known to exacerbate and propel neurodegeneration. IL-1β induces glial cells to produce cytokines and growth factors consistent with continued neuroinflammation [117] (see also Figure 1D). It is plausible that elevated IL-1β and other proinflammatory cytokines lead to a secondary effect on the BBB and allow entry into the brain of virus particles via alterations in BBB permeability, even though this particular scenario has not been investigated to date using experimental models.

In sum, the mechanisms involved in COVID-19 effects on the brain are highly complex and may be caused either by direct or indirect systemic responses to the virus, as outlined in Figure 1. Disruption of the BBB and the recruitment of innate immune cells from the periphery, as well as vascular events in the brain, contribute to the neurological symptoms observed. Due to the high mutagenicity of the SARS-CoV-2 virus, continued studies into the mechanisms and routes of infection to the brain are warranted since the mechanisms and neurological symptoms might be altered as the virus evolves and after vaccination.

### 6.3. Transportation of the SARS-CoV-2 vs. in Exosomes

A crucial finding was recently reported, showing that the virus spreads from cell to cell—or organ to organ—in the body via exosomes that are released from infected cells [118,119]. Exosomes are small extracellular vesicles (EVs) that are formed within all cell types in the multivesicular body and then expunged from the cell with the purpose of getting rid of toxic or unneeded materials, as well as signaling to other cells in the vicinity [120]. We and others have shown that EVs also serve as excellent sources for biomarkers in the brain when purified EVs from the blood are further purified to contain only glial- or neuron-derived exosomes [121,122,123].

**Figure 2:**
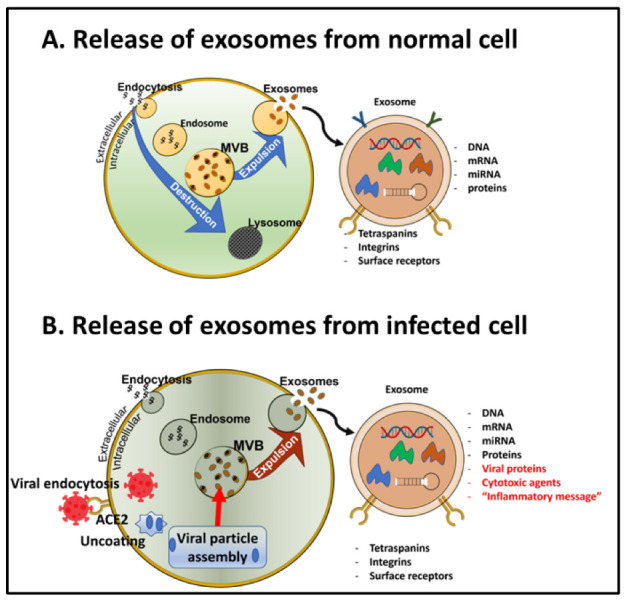
**Exosomes and viral trafficking. A = Release of exosomes from a normal cell. B = Release of exosomes from a SARS-CoV-2 infected cell**. (**A**) Small extracellular vesicles (EVs) are formed in the multivesicular body (MVB) in all cell types and then either destroyed in the autophagosomal pathway or released into extracellular space. The exosomes can either have a constitutive or activated release. An important role for EVs is to serve as an intercellular communication tool. (**B**) Exosomes purified from patients with SARS-CoV-2 contain viral particles and contribute both to the spread of infection and the adaptive immune response. Once in the cytoplasm, replication followed by viral particle assembly occurs. New membranous structures of various sizes and shapes are formed, referred to as “replication organelles”. These are transported to MVBs and incorporated into exosomes. Exosomes from COVID-19 patients contain viral proteins, cytotoxic agents, and an “inflammatory message” that is reactivated in the recipient cell.

Exosomes constitute a reliable route for misfolded protein transmission, contributing to the pathogenesis of neurodegenerative conditions such as PD and AD [100]. Travelling from the blood via the BBB into the brain parenchyma, exosomal cargo contains SARS-CoV-2 RNA, viral proteins, and inflammatory mediators but also modified host proteins that could promote neurodegenerative and neuroinflammatory cascades in the brain, potentially leading to the progression or acceleration of neurodegeneration in affected patients (see Figure 2). Thus, exosomes are produced by virus-infected cells and play an important role in mediating communication between infected and uninfected cells, most likely also within the brain and spinal cord. In addition, a recent manuscript by Gurunathan et al. [124] suggested that SARS-CoV-2 can modulate both the production and composition of exosomes and therefore exploit the biogenesis, secretion, and uptake of EVs to promote transmission of the infection to other cells. This group also proposed that exosomes loaded with immunomodulatory cargo could provide novel drug targets for COVID-19. Mysiris et al. posed that exosomes are involved in several different viral infections and that exosomes may contain cargo carrying both amyloidogenic proteins and viral particles from the periphery into the brain [100]. Exosomes released from infected cells contain misfolded proteins, including alpha-synuclein and p-Tau, that could propagate neurodegenerative prion-like proteins from brain region to brain region [100].

The role of exosomes for the transmission of viruses could be exploited for antiviral drug and vaccine development. Exosomes can be released either via stimulated or constitutive pathways (see Figure 2). Since there are drugs, for example, cancer drugs, that affect the release or uptake of exosomes [125], these might also potentially benefit patients with COVID-19 at the risk of developing neurodegenerative disorders. In addition, an exosomal analysis of blood can detect exosomal biomarkers, including pathogens, decades before any symptoms of cognitive or motor impairment are evident, providing an opportunity for preventative treatment [120,121,122,123,126,127,128,129,130,131]. The discovery that SARS-CoV-2 exerts many of its detrimental effects on the brain via exosome transport may therefore lead to better biomarkers for long COVID, novel treatment options, and examination of its role in neurodegenerative conditions.

## 7. Mechanisms for Down Syndrome-Related Vulnerability

In the beginning of the pandemic, it became evident that individuals with Down syndrome (DS) exhibited especially severe symptoms with COVID-19, with a 4-times higher risk of hospitalization, and were also subject to a significant increase in mortality with the disease (a 10-times higher risk (see, e.g., [132,133,134])). DS is associated with cardiovascular conditions [135,136,137], poor response to respiratory infections [138], and a reduced response to vaccination [139]. Respiratory distress is the most common cause of death in both children and adults with DS. The reduced number and maturation defects of T- and B-lymphocytes contribute to altered immunoglobulin levels, poor responses to vaccinations, and an increased severity of respiratory infections in children and adults with DS. These findings lead to an early recognition that those with DS had high priority for a vaccination against COVD-19.

The biological mechanisms involved in the vulnerability to COVID-19 include a severe immune dysregulation in DS [133,139,140], as well as the presence of the gene coding for TMPRSS2 (see above) on Chr. 21 [141]. In addition, four out of six known interferon receptors are also coded on Chr. 21 and are thought to contribute to the severe effects of viral infections in DS [133]. The superoxide dismutase-1 (SOD-1) gene is also located on Chr. 21 and contributes to the increased levels of oxidative stress throughout life in many tissues, including the brain [142].

Interestingly, there are several microRNAs (miRNA) on Chr. 21 that may affect the severity of infection for people with DS [143]. These include miR-155, miR-802, miR-125b-2, let-7c, and miR-99a [143]. Of these, some are particularly interesting from an AD and the COVID-19 perspective. MiR-155 is involved in inflammation and the conversion of microglia to the M1 phenotype [144]. Other miRNAs located on Chr. 21 are associated with cellular stress and AD pathology, including let-7c, miRNA-99a, miRNA125b, miRNA-155, and miRNA-802. Let-7c is elevated in patients with AD [145]. Even though we do not know yet which precise role miRNAs play in COVID-19 in the brain, several of these are located on Chr. 21 and may contribute to both the AD observed in nearly all persons with DS and the increased mortality of respiratory viral infections.

## 8. Conclusions and Next Steps

This review focused on the neurological symptoms of acute or long COVID and the biological mechanisms involved. The next steps in the research of the brain involvement of this novel coronavirus should involve (1) further exploration of the role of ACE and ACE2, as well as the angiotensins for the aggregation of misfolded proteins in neurodegeneration associated with the neurological consequences of COVID-19, (2) to identify effective treatment paradigms that may target ACE2 and/or other mechanisms involved in the increased severity of infection for AD patients and other vulnerable populations, and (3) to investigate the mechanisms of the exosome-related transportation of viral fragments within the brain and to the brain via cranial nerves and the vascular system. Exosome-regulating drugs could be used to limit the spread of infection in the brain, and, finally, (4) to include exosome cargo investigations when obtaining blood samples from COVID-19 patients to examine the biomarkers for specific brain-related post-acute sequelae of COVID-19. The mutability of the SARS-CoV-2 virus and its potential to directly affect the CNS highlights the urgency of developing technology to diagnose, manage, and treat brain injuries in COVID-19 patients. The emerging research on miRNAs and their role in the inflammatory cascade, especially in terms of spreading from cell to cell, might reveal their role in COVID-19, as well as dementia and neuroinflammation.

Limitations: One important factor that has not been discussed in this review is the mutation rate of the virus and the importance of that for future neurological implications of COVID-19. Will we see more or less cranial involvement in coronavirus infections with new mutations, and can these be mitigated by continued vaccinations? SARS-CoV-2 has been shown to have a high transmission rate in humans. It has been described previously that coronaviruses undergo modifications at a rate of 104 substitutions in a year per site. The mutation rates for the SARS-CoV and MERS-CoV whole genomes are assessed to be 0.80–2.38 × 10^3^ and 1.12 × 10^3^ nucleotide substitutions per year at a single site [146], meaning that we will most likely see variants of this and other coronaviruses for decades to come. What has been lacking in the field is also the systematic assessment of neuropathological sequalae for long COVID in the brain. As autopsy materials become available, more studies are sure to come. Vascular imaging and neuropathological analyses are needed to establish the correlations among virus-induced vascular disease, vasculitis, and COVID-19-related cerebrovascular and neurological syndromes.

## Data Availability

Not required since this is a review (no data included).

## References

[1] Shereen M.A., Khan S., Kazmi A., Bashir N., Siddique R. (2020). COVID-19 infection: Origin, transmission, and characteristics of human coronaviruses. J. Adv. Res..

[2] De Lusignan S., Lopez Bernal J., Zambon M., Akinyemi O., Amirthalingam G., Andrews N., Borrow R., Byford R., Charlett A., Dabrera G. (2020). Emergence of a Novel Coronavirus (COVID-19): Protocol for Extending Surveillance Used by the Royal College of General Practitioners Research and Surveillance Centre and Public Health England. JMIR Public Health Surveill..

[3] Huang X., Wei F., Hu L., Wen L., Chen K. (2020). Epidemiology and Clinical Characteristics of COVID-19. Arch. Iran. Med..

[4] Kooraki S., Hosseiny M., Myers L., Gholamrezanezhad A. (2020). Coronavirus (COVID-19) Outbreak: What the Department of Radiology Should Know. J. Am. Coll. Radiol..

[5] Okba N.M.A., Muller M.A., Li W., Wang C., GeurtsvanKessel C.H., Corman V.M., Lamers M.M., Sikkema R.S., de Bruin E., Chandler F.D. (2020). Severe Acute Respiratory Syndrome Coronavirus 2-Specific Antibody Responses in Coronavirus Disease Patients. Emerg. Infect. Dis..

[6] Palacios Cruz M., Santos E., Velazquez Cervantes M.A., Leon Juarez M. (2020). COVID-19, a worldwide public health emergency. Rev. Clin. Esp..

[7] Wouk J., Rechenchoski D.Z., Rodrigues B.C.D., Ribelato E.V., Faccin-Galhardi L.C. (2021). Viral infections and their relationship to neurological disorders. Arch. Virol..

[8] De Chiara G., Marcocci M.E., Sgarbanti R., Civitelli L., Ripoli C., Piacentini R., Garaci E., Grassi C., Palamara A.T. (2012). Infectious agents and neurodegeneration. Mol. Neurobiol..

[9] Bagetta G., Nistico G., Bowery N.G. (1990). Prevention by the NMDA receptor antagonist, MK801 of neuronal loss produced by tetanus toxin in the rat hippocampus. Br. J. Pharmacol..

[10] Zhou L., Miranda-Saksena M., Saksena N.K. (2013). Viruses and neurodegeneration. Virol. J..

[11] Majde J.A. (2010). Neuroinflammation resulting from covert brain invasion by common viruses—A potential role in local and global neurodegeneration. Med. Hypotheses.

[12] Kirtipal N., Kumar S., Dubey S.K., Dwivedi V.D., Gireesh Babu K., Maly P., Bharadwaj S. (2022). Understanding on the possible routes for SARS-CoV-2 invasion via ACE2 in the host linked with multiple organs damage. Infect. Genet. Evol..

[13] Chen R., Wang K., Yu J., Howard D., French L., Chen Z., Wen C., Xu Z. (2020). The Spatial and Cell-Type Distribution of SARS-CoV-2 Receptor ACE2 in the Human and Mouse Brains. Front. Neurol..

[14] Ding Q., Shults N.V., Gychka S.G., Harris B.T., Suzuki Y.J. (2021). Protein Expression of Angiotensin-Converting Enzyme 2 (ACE2) is Upregulated in Brains with Alzheimer’s Disease. Int. J. Mol. Sci..

[15] Hernandez V.S., Zetter M.A., Guerra E.C., Hernandez-Araiza I., Karuzin N., Hernandez-Perez O.R., Eiden L.E., Zhang L. (2021). ACE2 expression in rat brain: Implications for COVID-19 associated neurological manifestations. Exp. Neurol..

[16] Qiao J., Li W., Bao J., Peng Q., Wen D., Wang J., Sun B. (2020). The expression of SARS-CoV-2 receptor ACE2 and CD147, and protease TMPRSS2 in human and mouse brain cells and mouse brain tissues. Biochem. Biophys. Res. Commun..

[17] Clough E., Inigo J., Chandra D., Chaves L., Reynolds J.L., Aalinkeel R., Schwartz S.A., Khmaladze A., Mahajan S.D. (2021). Mitochondrial Dynamics in SARS-CoV-2 Spike Protein Treated Human Microglia: Implications for Neuro-COVID. J. Neuroimmune Pharmacol..

[18] Zhang Y., Chen X., Jia L., Zhang Y. (2022). Potential mechanism of SARS-CoV-2-associated central and peripheral nervous system impairment. Acta Neurol. Scand..

[19] Lindskog C., Mear L., Virhammar J., Fallmar D., Kumlien E., Hesselager G., Casar-Borota O., Rostami E. (2022). Protein Expression Profile of ACE2 in the Normal and COVID-19-Affected Human Brain. J. Proteome Res..

[20] Jeong G.U., Lyu J., Kim K.D., Chung Y.C., Yoon G.Y., Lee S., Hwang I., Shin W.H., Ko J., Lee J.Y. (2022). SARS-CoV-2 Infection of Microglia Elicits Proinflammatory Activation and Apoptotic Cell Death. Microbiol. Spectr..

[21] Netland J., Meyerholz D.K., Moore S., Cassell M., Perlman S. (2008). Severe acute respiratory syndrome coronavirus infection causes neuronal death in the absence of encephalitis in mice transgenic for human ACE2. J. Virol..

[22] Fernandez-Castaneda A., Lu P., Geraghty A.C., Song E., Lee M.H., Wood J., O’Dea M.R., Dutton S., Shamardani K., Nwangwu K. (2022). Mild respiratory COVID can cause multi-lineage neural cell and myelin dysregulation. Cell.

[23] Bilinska K., Jakubowska P., Von Bartheld C.S., Butowt R. (2020). Expression of the SARS-CoV-2 Entry Proteins, ACE2 and TMPRSS2, in Cells of the Olfactory Epithelium: Identification of Cell Types and Trends with Age. ACS Chem. Neurosci..

[24] Burks S.M., Rosas-Hernandez H., Alenjandro Ramirez-Lee M., Cuevas E., Talpos J.C. (2021). Can SARS-CoV-2 infect the central nervous system via the olfactory bulb or the blood-brain barrier?. Brain Behav. Immun..

[25] Abbasi A.Z., Kiyani D.A., Hamid S.M., Saalim M., Fahim A., Jalal N. (2021). Spiking dependence of SARS-CoV-2 pathogenicity on TMPRSS2. J. Med. Virol..

[26] Hoffmann M., Kleine-Weber H., Schroeder S., Kruger N., Herrler T., Erichsen S., Schiergens T.S., Herrler G., Wu N.H., Nitsche A. (2020). SARS-CoV-2 Cell Entry Depends on ACE2 and TMPRSS2 and Is Blocked by a Clinically Proven Protease Inhibitor. Cell.

[27] Wettstein L., Kirchhoff F., Munch J. (2022). The Transmembrane Protease TMPRSS2 as a Therapeutic Target for COVID-19 Treatment. Int. J. Mol. Sci..

[28] Hinduja A., Moutairou A., Calvet J.H. (2021). Sudomotor dysfunction in patients recovered from COVID-19. Neurophysiol. Clin..

[29] Pizzanelli C., Milano C., Canovetti S., Tagliaferri E., Turco F., Verdenelli S., Nesti L., Franchi M., Bonanni E., Menichetti F. (2021). Autoimmune limbic encephalitis related to SARS-CoV-2 infection: Case-report and review of the literature. Brain Behav. Immun. Health.

[30] Emmerton D., Abdelhafiz A.H. (2021). Care for Older People with Dementia During COVID-19 Pandemic. SN Compr. Clin. Med..

[31] Yu Y., Travaglio M., Popovic R., Leal N.S., Martins L.M. (2021). Alzheimer’s and Parkinson’s Diseases Predict Different COVID-19 Outcomes: A UK Biobank Study. Geriatrics.

[32] Yong S.J., Yong M.H., Teoh S.L., Soga T., Parhar I., Chew J., Lim W.L. (2021). The Hippocampal Vulnerability to Herpes Simplex Virus Type I Infection: Relevance to Alzheimer’s Disease and Memory Impairment. Front. Cell. Neurosci..

[33] Readhead B., Haure-Mirande J.V., Funk C.C., Richards M.A., Shannon P., Haroutunian V., Sano M., Liang W.S., Beckmann N.D., Price N.D. (2018). Multiscale Analysis of Independent Alzheimer’s Cohorts Finds Disruption of Molecular, Genetic, and Clinical Networks by Human Herpesvirus. Neuron.

[34] Galeotti C., Bayry J. (2020). Autoimmune and inflammatory diseases following COVID-19. Nat. Rev. Rheumatol..

[35] Al-Beltagi M., Saeed N.K., Bediwy A.S. (2022). COVID-19 disease and autoimmune disorders: A mutual pathway. World J. Methodol..

[36] Bhagavati S. (2021). Autoimmune Disorders of the Nervous System: Pathophysiology, Clinical Features, and Therapy. Front. Neurol..

[37] Rodriguez Y., Novelli L., Rojas M., De Santis M., Acosta-Ampudia Y., Monsalve D.M., Ramirez-Santana C., Costanzo A., Ridgway W.M., Ansari A.A. (2020). Autoinflammatory and autoimmune conditions at the crossroad of COVID-19. J. Autoimmun..

[38] Tufan A., Avanoglu Guler A., Matucci-Cerinic M. (2020). COVID-19, immune system response, hyperinflammation and repurposing antirheumatic drugs. Turk. J. Med. Sci..

[39] Al-Hazmi A. (2016). Challenges presented by MERS corona virus, and SARS corona virus to global health. Saudi J. Biol. Sci..

[40] Verstrepen K., Baisier L., De Cauwer H. (2020). Neurological manifestations of COVID-19, SARS and MERS. Acta Neurol. Belg..

[41] Anwar M.M., Badawi A.M., Eltablawy N.A. (2020). Can the coronavirus infection penetrates the brain resulting in sudden anosmia followed by severe neurological disorders?. eNeurologicalSci.

[42] Desforges M., Le Coupanec A., Dubeau P., Bourgouin A., Lajoie L., Dube M., Talbot P.J. (2019). Human Coronaviruses and Other Respiratory Viruses: Underestimated Opportunistic Pathogens of the Central Nervous System?. Viruses.

[43] Ibrahim Fouad G. (2021). The neuropathological impact of COVID-19: A review. Bull. Natl. Res. Cent..

[44] Wu Y., Xu X., Chen Z., Duan J., Hashimoto K., Yang L., Liu C., Yang C. (2020). Nervous system involvement after infection with COVID-19 and other coronaviruses. Brain Behav. Immun..

[45] Cavallieri F., Sellner J., Zedde M., Moro E. (2022). Neurologic complications of coronavirus and other respiratory viral infections. Handb. Clin. Neurol..

[46] McEntire C.R.S., Song K.W., McInnis R.P., Rhee J.Y., Young M., Williams E., Wibecan L.L., Nolan N., Nagy A.M., Gluckstein J. (2021). Neurologic Manifestations of the World Health Organization’s List of Pandemic and Epidemic Diseases. Front. Neurol..

[47] Meinhardt J., Radke J., Dittmayer C., Franz J., Thomas C., Mothes R., Laue M., Schneider J., Brunink S., Greuel S. (2021). Olfactory transmucosal SARS-CoV-2 invasion as a port of central nervous system entry in individuals with COVID-19. Nat. Neurosci..

[48] Harapan B.N., Yoo H.J. (2021). Neurological symptoms, manifestations, and complications associated with severe acute respiratory syndrome coronavirus 2 (SARS-CoV-2) and coronavirus disease 19 (COVID-19). J. Neurol..

[49] Maiese A., Manetti A.C., Bosetti C., Del Duca F., La Russa R., Frati P., Di Paolo M., Turillazzi E., Fineschi V. (2021). SARS-CoV-2 and the brain: A review of the current knowledge on neuropathology in COVID-19. Brain Pathol..

[50] Pajo A.T., Espiritu A.I., Apor A., Jamora R.D.G. (2021). Neuropathologic findings of patients with COVID-19: A systematic review. Neurol. Sci..

[51] Thakur K.T., Miller E.H., Glendinning M.D., Al-Dalahmah O., Banu M.A., Boehme A.K., Boubour A.L., Bruce S.S., Chong A.M., Claassen J. (2021). COVID-19 neuropathology at Columbia University Irving Medical Center/New York Presbyterian Hospital. Brain.

[52] Arbour N., Day R., Newcombe J., Talbot P.J. (2000). Neuroinvasion by human respiratory coronaviruses. J. Virol..

[53] Hui D.S.C., Zumla A. (2019). Severe Acute Respiratory Syndrome: Historical, Epidemiologic, and Clinical Features. Infect. Dis. Clin. North. Am..

[54] Pormohammad A., Ghorbani S., Khatami A., Farzi R., Baradaran B., Turner D.L., Turner R.J., Bahr N.C., Idrovo J.P. (2020). Comparison of confirmed COVID-19 with SARS and MERS cases—Clinical characteristics, laboratory findings, radiographic signs and outcomes: A systematic review and meta-analysis. Rev. Med. Virol..

[55] Mahase E. (2020). Coronavirus COVID-19 has killed more people than SARS and MERS combined, despite lower case fatality rate. BMJ.

[56] Guedj E., Campion J.Y., Dudouet P., Kaphan E., Bregeon F., Tissot-Dupont H., Guis S., Barthelemy F., Habert P., Ceccaldi M. (2021). (18)F-FDG brain PET hypometabolism in patients with long COVID. Eur. J. Nucl. Med. Mol. Imaging.

[57] Grossberg A.N., Koza L.A., Ledreux A., Prusmack C., Krishnamurthy H.K., Jayaraman V., Granholm A.C., Linseman D.A. (2021). A multiplex chemiluminescent immunoassay for serological profiling of COVID-19-positive symptomatic and asymptomatic patients. Nat. Commun..

[58] Mirfazeli F.S., Sarabi-Jamab A., Jahanbakhshi A., Kordi A., Javadnia P., Shariat S.V., Aloosh O., Almasi-Dooghaee M., Faiz S.H.R. (2020). Neuropsychiatric manifestations of COVID-19 can be clustered in three distinct symptom categories. Sci. Rep..

[59] Sweid A., Hammoud B., Bekelis K., Missios S., Tjoumakaris S.I., Gooch M.R., Herial N.A., Zarzour H., Romo V., DePrince M. (2020). Cerebral ischemic and hemorrhagic complications of coronavirus disease 2019. Int. J. Stroke.

[60] Aghayari Sheikh Neshin S., Shahjouei S., Koza E., Friedenberg I., Khodadadi F., Sabra M., Kobeissy F., Ansari S., Tsivgoulis G., Li J. (2021). Stroke in SARS-CoV-2 Infection: A Pictorial Overview of the Pathoetiology. Front. Cardiovasc. Med..

[61] Mutiawati E., Fahriani M., Mamada S.S., Fajar J.K., Frediansyah A., Maliga H.A., Ilmawan M., Emran T.B., Ophinni Y., Ichsan I. (2021). Anosmia and dysgeusia in SARS-CoV-2 infection: Incidence and effects on COVID-19 severity and mortality, and the possible pathobiology mechanisms—A systematic review and meta-analysis. F1000Research.

[62] Pua Torrejon R.C., Ordono Saiz M.V., Gonzalez Alguacil E., Furones Garcia M., Cantarin Extremera V., Ruiz Falco M.L., Soto Insuga V. (2022). Smell and Taste Dysfunction in Pediatric Patients With SARS-CoV-2 Infection. Pediatr. Neurol..

[63] Ortiz-Prado E., Simbana-Rivera K., Gomez-Barreno L., Rubio-Neira M., Guaman L.P., Kyriakidis N.C., Muslin C., Jaramillo A.M.G., Barba-Ostria C., Cevallos-Robalino D. (2020). Clinical, molecular, and epidemiological characterization of the SARS-CoV-2 virus and the Coronavirus Disease 2019 (COVID-19), a comprehensive literature review. Diagn. Microbiol. Infect. Dis..

[64] Chen Y., Yang W., Chen F., Cui L. (2022). COVID-19 and cognitive impairment: Neuroinvasive and blood–brain barrier dysfunction. J. Neuroinflammation.

[65] Tyson B., Shahein A., Erdodi L., Tyson L., Tyson R., Ghomi R., Agarwal P. (2022). Delirium as a Presenting Symptom of COVID-19. Cogn. Behav. Neurol..

[66] Caronna E., Ballve A., Llaurado A., Gallardo V.J., Ariton D.M., Lallana S., Lopez Maza S., Olive Gadea M., Quibus L., Restrepo J.L. (2020). Headache: A striking prodromal and persistent symptom, predictive of COVID-19 clinical evolution. Cephalalgia.

[67] Rahman S., Montero M.T.V., Rowe K., Kirton R., Kunik F. (2021). Epidemiology, pathogenesis, clinical presentations, diagnosis and treatment of COVID-19: A review of current evidence. Expert. Rev. Clin. Pharmacol..

[68] Rathore V., Galhotra A., Pal R., Sahu K.K. (2020). COVID-19 Pandemic and Children: A Review. J. Pediatr. Pharmacol. Ther..

[69] Rando H.M., Bennett T.D., Byrd J.B., Bramante C., Callahan T.J., Chute C.G., Davis H.E., Deer R., Gagnier J., Koraishy F.M. (2021). Challenges in defining Long COVID: Striking differences across literature, Electronic Health Records, and patient-reported information. medRxiv.

[70] Tana C., Bentivegna E., Cho S.J., Harriott A.M., Garcia-Azorin D., Labastida-Ramirez A., Ornello R., Raffaelli B., Beltran E.R., Ruscheweyh R. (2022). Long COVID headache. J. Headache Pain.

[71] Kakovan M., Ghorbani Shirkouhi S., Zarei M., Andalib S. (2022). Stroke Associated with COVID-19 Vaccines. J. Stroke Cerebrovasc. Dis..

[72] Kantarcioglu B., Iqbal O., Lewis J., Carter C.A., Singh M., Lievano F., Ligocki M., Jeske W., Adiguzel C., Gerotziafas G.T. (2022). An Update on the Status of Vaccine Development for SARS-CoV-2 Including Variants. Practical Considerations for COVID-19 Special Populations. Clin. Appl. Thromb. Hemost..

[73] Sriwastava S., Sharma K., Khalid S.H., Bhansali S., Shrestha A.K., Elkhooly M., Srivastava S., Khan E., Jaiswal S., Wen S. (2022). COVID-19 Vaccination and Neurological Manifestations: A Review of Case Reports and Case Series. Brain Sci..

[74] Bhandari B., Rayamajhi G., Lamichhane P., Shenoy A.K. (2022). Adverse Events following Immunization with COVID-19 Vaccines: A Narrative Review. Biomed. Res. Int..

[75] Pyne J.D., Brickman A.M. (2021). The Impact of the COVID-19 Pandemic on Dementia Risk: Potential Pathways to Cognitive Decline. Neurodegener. Dis..

[76] Barrantes F.J. (2020). Central Nervous System Targets and Routes for SARS-CoV-2: Current Views and New Hypotheses. ACS Chem. Neurosci..

[77] Deng Q., Rasool R.U., Russell R.M., Natesan R., Asangani I.A. (2021). Targeting androgen regulation of TMPRSS2 and ACE2 as a therapeutic strategy to combat COVID-19. iScience.

[78] Ni W., Yang X., Yang D., Bao J., Li R., Xiao Y., Hou C., Wang H., Liu J., Yang D. (2020). Role of angiotensin-converting enzyme 2 (ACE2) in COVID-19. Crit. Care.

[79] Urmila A., Rashmi P., Nilam G., Subhash B. (2021). Recent Advances in the Endogenous Brain Renin-Angiotensin System and Drugs Acting on It. J. Renin Angiotensin Aldosterone Syst..

[80] Wang X.L., Iwanami J., Min L.J., Tsukuda K., Nakaoka H., Bai H.Y., Shan B.S., Kan-No H., Kukida M., Chisaka T. (2016). Deficiency of angiotensin-converting enzyme 2 causes deterioration of cognitive function. NPJ Aging Mech. Dis..

[81] Liu S., Liu J., Miura Y., Tanabe C., Maeda T., Terayama Y., Turner A.J., Zou K., Komano H. (2014). Conversion of Abeta43 to Abeta40 by the successive action of angiotensin-converting enzyme 2 and angiotensin-converting enzyme. J. Neurosci. Res..

[82] Parit R., Jayavel S. (2021). Association of ACE inhibitors and Angiotensin type II blockers with ACE2 overexpression in COVID-19 comorbidities: A pathway-based analytical study. Eur. J. Pharmacol..

[83] Bennett R.E., Esparza T.J., Lewis H.A., Kim E., Mac Donald C.L., Sullivan P.M., Brody D.L. (2013). Human apolipoprotein E4 worsens acute axonal pathology but not amyloid-beta immunoreactivity after traumatic brain injury in 3xTG-AD mice. J. Neuropathol. Exp. Neurol..

[84] Chen F., Chen Y., Wang Y., Ke Q., Cui L. (2022). The COVID-19 pandemic and Alzheimer’s disease: Mutual risks and mechanisms. Transl. Neurodegener..

[85] Kurki S.N., Kantonen J., Kaivola K., Hokkanen L., Mayranpaa M.I., Puttonen H., FinnGen, Martola J., Poyhonen M., Kero M. (2021). APOE epsilon4 associates with increased risk of severe COVID-19, cerebral microhaemorrhages and post-COVID mental fatigue: A Finnish biobank, autopsy and clinical study. Acta Neuropathol. Commun..

[86] Peterson A., Marengoni A., Shenkin S., MacLullich A. (2021). Delirium in COVID-19: Common, distressing and linked with poor outcomes... can we do better?. Age Ageing.

[87] Voormolen D.C., Cnossen M.C., Polinder S., von Steinbuechel N., Vos P.E., Haagsma J.A. (2018). Divergent Classification Methods of Post-Concussion Syndrome after Mild Traumatic Brain Injury: Prevalence Rates, Risk Factors, and Functional Outcome. J. Neurotrauma.

[88] Bullock G.S., Emery C.A., Nelson V.R., Prats-Uribe A., Gilliland R.G., Thigpen C.A., Shanley E. (2023). Higher rates of concussion following COVID-19 infection in high school athletes. Br. J. Sport. Med..

[89] Bodnar C.N., Watson J.B., Higgins E.K., Quan N., Bachstetter A.D. (2021). Inflammatory Regulation of CNS Barriers After Traumatic Brain Injury: A Tale Directed by Interleukin-1. Front. Immunol..

[90] Welcome M.O., Mastorakis N.E. (2021). Neuropathophysiology of coronavirus disease 2019: Neuroinflammation and blood brain barrier disruption are critical pathophysiological processes that contribute to the clinical symptoms of SARS-CoV-2 infection. Inflammopharmacology.

[91] DeKosky S.T., Kochanek P.M., Valadka A.B., Clark R.S.B., Chou S.H., Au A.K., Horvat C., Jha R.M., Mannix R., Wisniewski S.R. (2021). Blood Biomarkers for Detection of Brain Injury in COVID-19 Patients. J. Neurotrauma.

[92] Shi K., Zhang J., Dong J.F., Shi F.D. (2019). Dissemination of brain inflammation in traumatic brain injury. Cell. Mol. Immunol..

[93] Fotuhi M., Mian A., Meysami S., Raji C.A. (2020). Neurobiology of COVID-19. J. Alzheimers Dis..

[94] Sikora E., Bielak-Zmijewska A., Dudkowska M., Krzystyniak A., Mosieniak G., Wesierska M., Wlodarczyk J. (2021). Cellular Senescence in Brain Aging. Front. Aging Neurosci..

[95] Mattson M.P., Arumugam T.V. (2018). Hallmarks of Brain Aging: Adaptive and Pathological Modification by Metabolic States. Cell Metab..

[96] Karnik M., Beeraka N.M., Uthaiah C.A., Nataraj S.M., Bettadapura A.D.S., Aliev G., Madhunapantula S.V. (2021). A Review on SARS-CoV-2-Induced Neuroinflammation, Neurodevelopmental Complications, and Recent Updates on the Vaccine Development. Mol. Neurobiol..

[97] Vargas G., Medeiros Geraldo L.H., Gedeao Salomao N., Viana Paes M., Regina Souza Lima F., Carvalho Alcantara Gomes F. (2020). Severe acute respiratory syndrome coronavirus 2 (SARS-CoV-2) and glial cells: Insights and perspectives. Brain Behav. Immun. Health.

[98] Mavrikaki M., Lee J.D., Solomon I.H., Slack F.J. (2021). Severe COVID-19 induces molecular signatures of aging in the human brain. medRxiv.

[99] Idrees D., Kumar V. (2021). SARS-CoV-2 spike protein interactions with amyloidogenic proteins: Potential clues to neurodegeneration. Biochem. Biophys. Res. Commun..

[100] Mysiris D.S., Vavougios G.D., Karamichali E., Papoutsopoulou S., Stavrou V.T., Papayianni E., Boutlas S., Mavridis T., Foka P., Zarogiannis S.G. (2022). Post-COVID-19 Parkinsonism and Parkinson’s Disease Pathogenesis: The Exosomal Cargo Hypothesis. Int. J. Mol. Sci..

[101] Groh N., Buhler A., Huang C., Li K.W., van Nierop P., Smit A.B., Fandrich M., Baumann F., David D.C. (2017). Age-Dependent Protein Aggregation Initiates Amyloid-beta Aggregation. Front. Aging Neurosci..

[102] Yan C.H., Prajapati D.P., Ritter M.L., DeConde A.S. (2020). Persistent Smell Loss Following Undetectable SARS-CoV-2. Otolaryngol. Head Neck Surg..

[103] Dell’Era V., Farri F., Garzaro G., Gatto M., Aluffi Valletti P., Garzaro M. (2020). Smell and taste disorders during COVID-19 outbreak: A cross-sectional study on 355 patients. Head Neck.

[104] Menni C., Valdes A.M., Freidin M.B., Sudre C.H., Nguyen L.H., Drew D.A., Ganesh S., Varsavsky T., Cardoso M.J., El-Sayed Moustafa J.S. (2020). Real-time tracking of self-reported symptoms to predict potential COVID-19. Nat. Med..

[105] Song E., Zhang C., Israelow B., Lu-Culligan A., Prado A.V., Skriabine S., Lu P., Weizman O.E., Liu F., Dai Y. (2021). Neuroinvasion of SARS-CoV-2 in human and mouse brain. J. Exp. Med..

[106] Xu J., Lazartigues E. (2020). Expression of ACE2 in Human Neurons Supports the Neuro-Invasive Potential of COVID-19 Virus. Cell. Mol. Neurobiol..

[107] Schuler B.A., Habermann A.C., Plosa E.J., Taylor C.J., Jetter C., Negretti N.M., Kapp M.E., Benjamin J.T., Gulleman P., Nichols D.S. (2021). Age-determined expression of priming protease TMPRSS2 and localization of SARS-CoV-2 in lung epithelium. J. Clin. Investig..

[108] Uversky V.N., Elrashdy F., Aljadawi A., Ali S.M., Khan R.H., Redwan E.M. (2020). Severe acute respiratory syndrome coronavirus 2 infection reaches the human nervous system: How?. J. Neurosci. Res..

[109] Kunnumakkara A.B., Rana V., Parama D., Banik K., Girisa S., Sahu H., Thakur K.K., Dutta U., Garodia P., Gupta S.C. (2021). COVID-19, cytokines, inflammation, and spices: How are they related?. Life Sci..

[110] Agrawal S., Farfel J.M., Arfanakis K., Al-Harthi L., Shull T., Teppen T.L., Evia A.M., Patel M.B., Ely E.W., Leurgans S.E. (2022). Brain autopsies of critically ill COVID-19 patients demonstrate heterogeneous profile of acute vascular injury, inflammation and age-linked chronic brain diseases. Acta Neuropathol. Commun..

[111] Plini E.R.G., O’Hanlon E., Boyle R., Sibilia F., Rikhye G., Kenney J., Whelan R., Melnychuk M.C., Robertson I.H., Dockree P.M. (2021). Examining the Role of the Noradrenergic Locus Coeruleus for Predicting Attention and Brain Maintenance in Healthy Old Age and Disease: An MRI Structural Study for the Alzheimer’s Disease Neuroimaging Initiative. Cells.

[112] Bocci M., Oudenaarden C., Saenz-Sarda X., Simren J., Eden A., Sjolund J., Moller C., Gisslen M., Zetterberg H., Englund E. (2021). Infection of Brain Pericytes Underlying Neuropathology of COVID-19 Patients. Int. J. Mol. Sci..

[113] Serrano G.E., Walker J.E., Arce R., Glass M.J., Vargas D., Sue L.I., Intorcia A.J., Nelson C.M., Oliver J., Papa J. (2021). Mapping of SARS-CoV-2 Brain Invasion and Histopathology in COVID-19 Disease. medRxiv.

[114] Quan D., Luna Wong L., Shallal A., Madan R., Hamdan A., Ahdi H., Daneshvar A., Mahajan M., Nasereldin M., Van Harn M. (2021). Impact of Race and Socioeconomic Status on Outcomes in Patients Hospitalized with COVID-19. J. Gen. Intern. Med..

[115] Maedler K., Dharmadhikari G., Schumann D.M., Storling J., Schwanstecher M. (2011). Interleukin-targeted therapy for metabolic syndrome and type 2 diabetes. Diabetes-Perspectives in Drug Therapy.

[116] Argaw A.T., Zhang Y., Snyder B.J., Zhao M.L., Kopp N., Lee S.C., Raine C.S., Brosnan C.F., John G.R. (2006). IL-1beta regulates blood-brain barrier permeability via reactivation of the hypoxia-angiogenesis program. J. Immunol..

[117] Friedman W.J. (2001). Cytokines regulate expression of the type 1 interleukin-1 receptor in rat hippocampal neurons and glia. Exp. Neurol..

[118] Lam S.M., Huang X., Shui G. (2022). Neurological aspects of SARS-CoV-2 infection: Lipoproteins and exosomes as Trojan horses. Trends Endocrinol. Metab..

[119] Pesce E., Manfrini N., Cordiglieri C., Santi S., Bandera A., Gobbini A., Gruarin P., Favalli A., Bombaci M., Cuomo A. (2021). Exosomes Recovered From the Plasma of COVID-19 Patients Expose SARS-CoV-2 Spike-Derived Fragments and Contribute to the Adaptive Immune Response. Front. Immunol..

[120] Barile L., Vassalli G. (2017). Exosomes: Therapy delivery tools and biomarkers of diseases. Pharmacol. Ther..

[121] DeLeo A.M., Ikezu T. (2018). Extracellular Vesicle Biology in Alzheimer’s Disease and Related Tauopathy. J. Neuroimmune Pharmacol..

[122] Hamlett E.D., Ledreux A., Potter H., Chial H.J., Patterson D., Espinosa J.M., Bettcher B.M., Granholm A.C. (2018). Exosomal biomarkers in Down syndrome and Alzheimer’s disease. Free Radic. Biol. Med..

[123] Hamlett E.D., Goetzl E.J., Ledreux A., Vasilevko V., Boger H.A., LaRosa A., Clark D., Carroll S.L., Carmona-Iragui M., Fortea J. (2016). Neuronal exosomes reveal Alzheimer’s disease biomarkers in Down syndrome. Alzheimers Dement..

[124] Gurunathan S., Kang M.H., Kim J.H. (2021). Diverse Effects of Exosomes on COVID-19: A Perspective of Progress From Transmission to Therapeutic Developments. Front. Immunol..

[125] Zhang H., Lu J., Liu J., Zhang G., Lu A. (2020). Advances in the discovery of exosome inhibitors in cancer. J. Enzym. Inhib. Med. Chem..

[126] Fiandaca M.S., Kapogiannis D., Mapstone M., Boxer A., Eitan E., Schwartz J.B., Abner E.L., Petersen R.C., Federoff H.J., Miller B.L. (2015). Identification of preclinical Alzheimer’s disease by a profile of pathogenic proteins in neurally derived blood exosomes: A case-control study. Alzheimers Dement..

[127] Goetzl E.J., Elahi F.M., Mustapic M., Kapogiannis D., Pryhoda M., Gilmore A., Gorgens K.A., Davidson B., Granholm A.C., Ledreux A. (2019). Altered levels of plasma neuron-derived exosomes and their cargo proteins characterize acute and chronic mild traumatic brain injury. FASEB J..

[128] Goetzl E.J., Mustapic M., Kapogiannis D., Eitan E., Lobach I.V., Goetzl L., Schwartz J.B., Miller B.L. (2016). Cargo proteins of plasma astrocyte-derived exosomes in Alzheimer’s disease. FASEB J..

[129] Guix F.X., Corbett G.T., Cha D.J., Mustapic M., Liu W., Mengel D., Chen Z., Aikawa E., Young-Pearse T., Kapogiannis D. (2018). Detection of Aggregation-Competent Tau in Neuron-Derived Extracellular Vesicles. Int. J. Mol. Sci..

[130] Kenney K., Qu B.X., Lai C., Devoto C., Motamedi V., Walker W.C., Levin H.S., Nolen T., Wilde E.A., Diaz-Arrastia R. (2018). Higher exosomal phosphorylated tau and total tau among veterans with combat-related repetitive chronic mild traumatic brain injury. Brain Inj..

[131] Li P., Kaslan M., Lee S.H., Yao J., Gao Z. (2017). Progress in Exosome Isolation Techniques. Theranostics.

[132] Clift A.K., Coupland C.A.C., Keogh R.H., Hemingway H., Hippisley-Cox J. (2020). COVID-19 Mortality Risk in Down Syndrome: Results From a Cohort Study Of 8 Million Adults. Ann. Intern. Med..

[133] Espinosa J.M. (2020). Down Syndrome and COVID-19: A Perfect Storm?. Cell. Rep. Med..

[134] Huls A., Feany P.T., Zisman S.I., Costa A.C.S., Dierssen M., Balogh R., Bargagna S., Baumer N.T., Brandao A.C., Carfi A. (2022). COVID-19 Vaccination of Individuals with Down Syndrome-Data from the Trisomy 21 Research Society Survey on Safety, Efficacy, and Factors Associated with the Decision to Be Vaccinated. Vaccines.

[135] Cooper S.A., Ademola T., Caslake M., Douglas E., Evans J., Greenlaw N., Haig C., Hassiotis A., Jahoda A., McConnachie A. (2016). Towards onset prevention of cognition decline in adults with Down syndrome (The TOP-COG study): A pilot randomised controlled trial. Trials.

[136] Real de Asua D., Parra P., Costa R., Moldenhauer F., Suarez C. (2014). A cross-sectional study of the phenotypes of obesity and insulin resistance in adults with down syndrome. Diabetes Metab. J..

[137] Wilcock D.M., Schmitt F.A., Head E. (2016). Cerebrovascular contributions to aging and Alzheimer’s disease in Down syndrome. Biochim. Biophys. Acta.

[138] Illouz T., Biragyn A., Iulita M.F., Flores-Aguilar L., Dierssen M., De Toma I., Antonarakis S.E., Yu E., Herault Y., Potier M.C. (2021). Immune Dysregulation and the Increased Risk of Complications and Mortality Following Respiratory Tract Infections in Adults With Down Syndrome. Front. Immunol..

[139] Colvin K.L., Yeager M.E. (2017). What people with Down Syndrome can teach us about cardiopulmonary disease. Eur. Respir. Rev..

[140] Satge D., Seidel M.G. (2018). The Pattern of Malignancies in Down Syndrome and Its Potential Context With the Immune System. Front. Immunol..

[141] Andolfo I., Russo R., Lasorsa V.A., Cantalupo S., Rosato B.E., Bonfiglio F., Frisso G., Abete P., Cassese G.M., Servillo G. (2021). Common variants at 21q22.3 locus influence MX1 and TMPRSS2 gene expression and susceptibility to severe COVID-19. iScience.

[142] Lockrow J.P., Fortress A.M., Granholm A.C. (2012). Age-related neurodegeneration and memory loss in down syndrome. Curr. Gerontol. Geriatr. Res..

[143] Bras A., Rodrigues A.S., Gomes B., Rueff J. (2018). Down syndrome and microRNAs. Biomed. Rep..

[144] Pasca S., Jurj A., Petrushev B., Tomuleasa C., Matei D. (2020). MicroRNA-155 Implication in M1 Polarization and the Impact in Inflammatory Diseases. Front. Immunol..

[145] Derkow K., Rossling R., Schipke C., Kruger C., Bauer J., Fahling M., Stroux A., Schott E., Ruprecht K., Peters O. (2018). Distinct expression of the neurotoxic microRNA family let-7 in the cerebrospinal fluid of patients with Alzheimer’s disease. PLoS ONE.

[146] Penarrubia A.L., Ruiz M., Porco R., Rao S.N., Juanola-Falgarona M., Manissero D., Lopez-Fontanals M., Pareja J. (2020). Multiple assays in a real-time RT-PCR SARS-CoV-2 panel can mitigate the risk of loss of sensitivity by new genomic variants during the COVID-19 outbreak. Int. J. Infect. Dis..

